# Sudden cardiac arrest in a child with Gitelman syndrome: a case report and literature review

**DOI:** 10.3389/fped.2023.1188098

**Published:** 2023-06-07

**Authors:** Jakub Zieg, Terezia Tavačová, Miroslava Balaščáková, Petra Peldová, Filip Fencl, Peter Kubuš

**Affiliations:** ^1^ Department of Pediatrics, Second Faculty of Medicine, Charles University and Motol University Hospital, Prague, Czechia; ^2^ Children's Heart Centre, 2nd Faculty of Medicine, Charles University and Motol University Hospital, Prague, Czechia; ^3^ Department of Biology and Medical Genetics, University Hospital Motol, Second Medical Faculty, Charles University, Prague, Czechia

**Keywords:** arrhythmia, Gitelman syndrome, hypokalemia, hypomagnesemia, sudden cardiac arrest

## Abstract

Salt-losing tubulopathies are well-recognised diseases predisposing to metabolic disturbances in affected patients. One of the most severe complications can be life-threatening arrhythmias causing sudden cardiac arrest. We present here the first case of a pediatric patient with Gitelman syndrome associated sudden cardiac arrest without precipitating event. A 10-year-old boy collapsed due to ventricular fibrillation in the Prague tram. Lay cardiopulmonary resuscitation was initiated and external defibrillation restored sinus rhythm within minutes. Initial laboratory examination revealed severe hypokalemia requiring large amounts of electrolyte supplementation. Genetic testing focused to tubulopathies was performed and the diagnosis of Gitelman syndrome was made following the identification of two pathogenic variants in *SLC12A3* gene (c.2633 + 1G>A and c.2221G>A). Implantable cardioverter-defibrillator was implanted to prevent sudden cardiac death. The patient was in a good clinical condition with satisfactory electrolyte serum levels at the last follow-up. Causes of electrolyte abnormalities in children should be identified early to prevent the development of rare but potentially fatal complications.

## Background

Gitelman syndrome (GS) is an autosomal recessive salt-losing tubulopathy characterized by hypokalemic metabolic alkalosis, hypomagnesemia, hypocalciuria and secondary hyperaldosteronism. This disease was first described in three individuals (two sisters and one unrelated patient) by Gitelman, Graham and Welt in 1966 (1). GS represents the most common hereditary tubulopathy with prevalence estimated at 25 per million (2). GS is caused by homozygous or compound heterozygous mutations in the *SLC12A3* gene, which encodes the thiazide-sensitive sodium-chloride cotransporter (NCC), that is expressed in the apical membrane of cells in the distal convoluted tubule (3). GS metabolic abnormalities mimic treatment with thiazide diuretics, which act by blocking this tubular channel. The first symptoms usually present in children older than 6 years or in adolescence or adulthood, but cases of neonatal presentation have also been reported (4). The most common clinical manifestations include salt craving, muscle weakness, cramps, fatigue, dizziness, nocturia, polydipsia, paraesthesia, numbness, palpitations and hypotension (5). Genetic testing should be offered to all affected individuals with a clinical suspicion of GS. The mainstay of therapy is supplementation of electrolytes, some cases require further medication to maintain satisfactory electrolyte serum concentration. Although electrolyte abnormalities in children with salt-losing tubulopathies may be asymptomatic, severe life threatening cardiac arrhythmias have been reported in children with Bartter syndrome, a hereditary tubulopathy affecting the transport mechanisms in the loop of Henle with phenotype that can in certain forms resemble GS (6). Interestingly, prolonged corrected QT interval associated with the risk of fatal arrhythmias was found in a cohort patients with GS (7). We present here a case of ventricular fibrillation causing sudden cardiac arrest (SCA) in a child with GS.

## Case description

**Figure 1:**
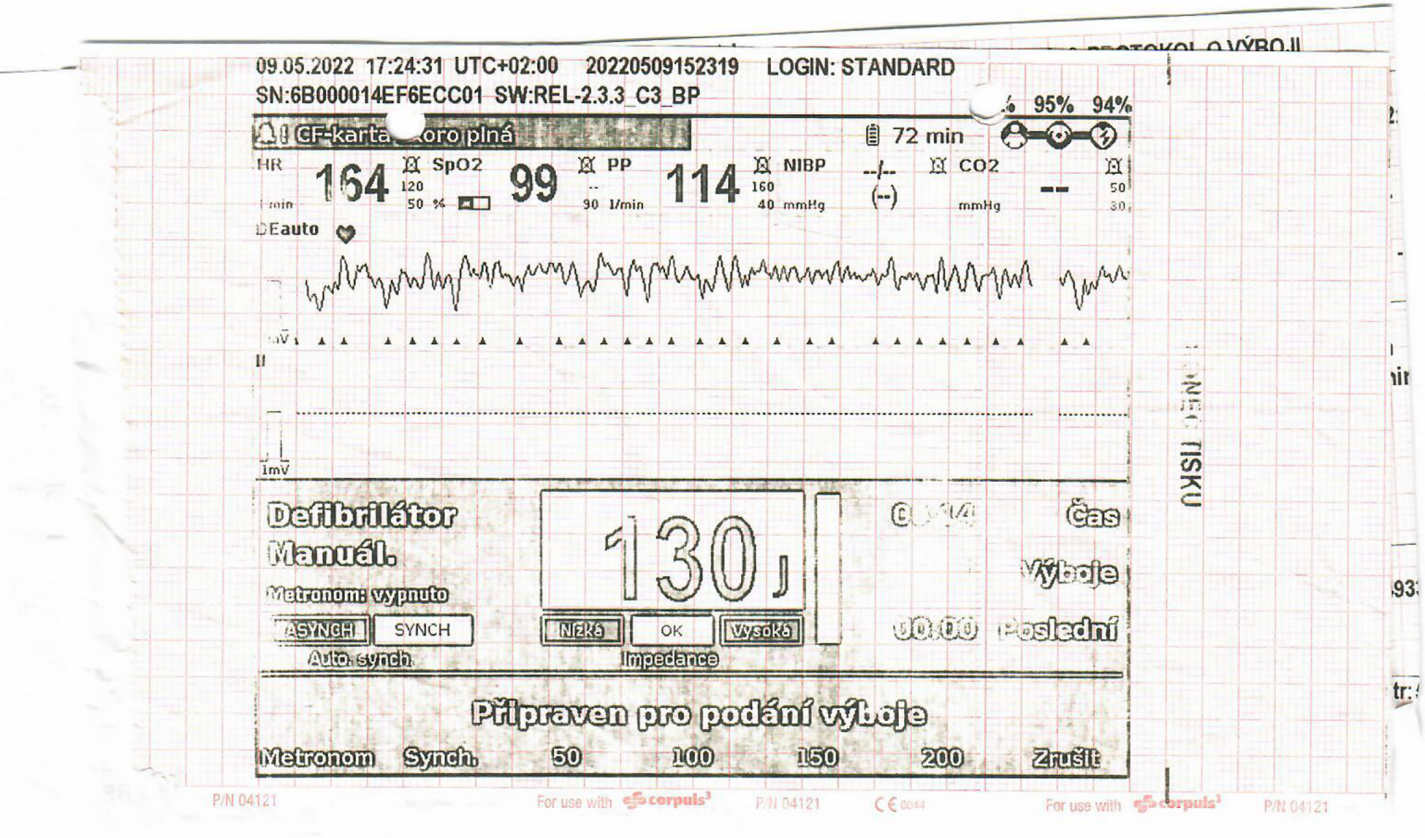
ECG during sudden cardiac arrest with documented ventricular fibrillation.

**Figure 2:**
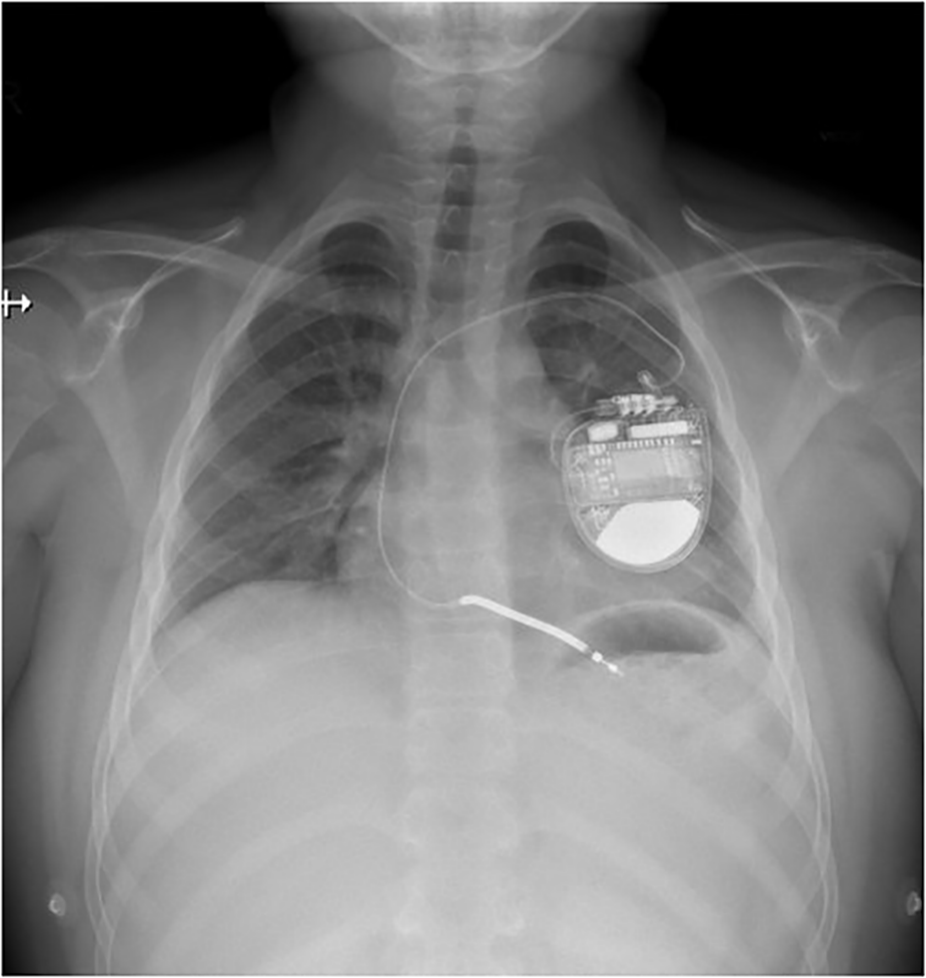
Transvenous implantable cardioverter-defibrillator implanted in patient with GS.

10-year-old boy (refugee from Ukraine) collapsed in the tram in the city of Prague. Lay cardiopulmonary resuscitation was initiated with subsequent arrival of healthcare professionals. Sinus rhythm was restored within a few minutes by external defibrillation of the documented ventricular fibrillation (Figure 1). The patient was intubated, sedated and transported to the pediatric anesthesiologic ward, with no major physical findings except for pathologic lung auscultation—crackling and wheezing over the right lung caused by aspiration pneumonia, managed by parenteral antibiotic therapy (amoxycilin/clavulanate). On admission, the patient was stable under sedation, intubated with no reaction to examination (Glasgow coma scale 3). Therapeutic hypothermia was slowly withdrawn after 24 h. Electrocardiogram (ECG) showed normal sinus rhythm with age-appropriate heart rate and normal repolarization, with QTc of 422 ms (including normal QTc during 24 h ECG monitoring) and normal echocardiography findings. The initial laboratory results were as follows: serum sodium 133 mmol/L, potassium 2.5 mmol/L, chloride 96 mmol/L, calcium 2.25 mmol/L, magnesium 0.78 mmol/L, urea 7.4 mmol/L, creatinine 66 µmol/L, bicarbonate 22.9 mmol/L. Parenteral electrolyte supplementation was started, high potassium chloride (KCl) supplementation 8–10 mmol/kg/day was required to maintain potassium in the low normal range, magnesium was supplemented later when serum levels were decreased. The patient was extubated on the second day with normal neurologic examination and transported to Children's Heart Centre on the 4th day of hospitalization. Oral celecoxib (3.5 mg/kg/day) was started after 7 days to reduce the extensive oral and parenteral KCl supplementation. Single-chamber (transvenous lead) implantable cardioverter-defibrillator (ICD) was implanted for secondary prevention of sudden cardiac death (Figure 2). The patient was discharged from the hospital after 28 days. He has been followed up regularly by a pediatric nephrologist and cardiologist, with satisfactory potassium and magnesium serum levels in the long term, >3 mmol/L and >0.6 mmol/L, respectively. Since the device implantation, the remote ICD monitoring recorded several episodes of asymptomatic non-sustained polymorphic ventricular tachycardia, with no appropriate therapies needed so far. Molecular genetic examination performed by next-generation sequencing (Clinical Exome Solution v3 by Sophia Genetics, Switzerland) revealed 2 pathogenic (class 5 according to ACMG/AMP guidelines) variants in *SLC12A3* gene (NM_001126108) c.2633 + 1G>A and c.2221G>A (p.Gly741Arg) confirming the diagnosis of GS. Subsequent segregation analysis revealed 2 pathogenic (class 5 according to ACMG/AMP guidelines) variants in *SLC12A3* gene (NM_001126108) paternally inherited c.2633 + 1G>A and maternally inherited c.2221G>A (p.Gly741Arg).

## Discussion

Cardiac arrhythmias and SCA are rare and severe complications of salt-losing tubulopathies. Electrolyte depletion and/or QTc prolongation are presumed factors involved in the pathogenesis of these heart conduction disorders (8). Although these patients are usually adapted to chronic hypokalemia, certain clinical conditions such as vomiting and diarrhea may further lower serum potassium concentration and induce arrhythmia (6). Interestingly, our patient did not have any clinical signs of viral illness or gastrointestinal symptoms at the time of cardiac event. Although literature on cardiac evaluation of GS patients is scarce, a significant proportion of GS patients have been found to have long QTc, therefore, thorough cardiac work up is warranted in these individuals (7, 9). Standard management of GS patients consists of electrolyte supplementation to achieve targets for potassium 3 mmol/L and for magnesium 0.6 mmol/L. This therapy may be complicated by mainly gastrointestinal side effects, therefore, in some patients the accepted electrolyte concentration may be lower. Nonsteroidal anti-inflammatory drugs, potassium sparing diuretics and inhibitors of renin-angiotensin-aldosteron axis (RAAA) may be considered in children with persistent hypokalemia despite high doses of supplementation, unacceptable side effects and good adherence to therapy. Hypomagnesemia may have characteristic ECG pattern, especially prologantion of QTc interval associated with risk of severe ventricular arrhythmia (polymorphic ventricular tachycardia/torsades de pointes, ventricular fibrillation) (10). Flat or absent T-waves, ST segment depression and U waves may be identified on ECG in patients with moderately to severely decreased potassium serum concentration (11). In the reported patient, no such repolarization changes were observed, which suggests highly variable effect of electrolyte imbalance on ECG findings in different individuals. Hypokalemia causes resting membrane hyperpolarization, Na^+^/K^+^ ATPase inhibition, Na^+^ and Ca^2+^ intracellular accumulation with reduced repolarization reserve, that predisposes to early- and delayed-afterdepolarization mediated arrhythmias (12). Potassium serum levels may decrease in conditions with volume depletion, which activates RAAA (13). Therefore, in case of dehydration, children with salt-losing nephropathies should be early referred and treated with isotonic saline and increased electrolyte (potassium with or without concurrent magnesium) supplementation. Interestingly, magnesium influences the function of Na+/K+ATPase and it's depletion leads to hypokalemia. Cortessi et al. performed survey research among European pediatric kidney disease specialists treating Bartter syndrome and GS. 249 patients were included in this study. They aimed to study the frequency and character of cardiac arrhythmias and rhabdomyolysis in children with salt-losing tubulopathies. Seven children (2.8%) with acute cardiac complications were found, only one of these individuals was diagnosed with GS. Three children including the child with GS suffered from diarrhea or vomiting as a supposed precipitating factor (14). The indication for ICD implantation was thoroughly discussed—the combination of previous nearly fatal arrhythmia (i.e., secondary prevention) and (at least) potential for arrhythmia recurrence in case of suboptimal electrolyte management (which makes the idea of “reversible cause” of sudden cardiac death at least discutable), led us to the decision to implant the device. Indication for ICD in children is less clearly established and, in our Centre, at present, we prefer transvenous ICD in secondary prevention. Moreover, recurrent findings of non-sustained polymorphic ventricular tachycardia after ICD implantation support our decision to implant.

Although there is a published case report of adult with Gitelman syndrome presenting with cardiac arrest (15), to our knowledge, we present the first case of SCA in a child with GS without any precipitating event. Appropriate assessment of serum and urinary concentrations of electrolytes including magnesium is indicated in patients with VA. Salt-losing nephropathies represent one of the most common ethiology of hypokalemia and hypomagnesemia. The risk of arrhythmias in these children may be increased in certain clinical situations (e.g., dehydration, drugs). Clinicians should be aware that rare life-threatening cardiac events can occur in these individuals.

## Data Availability

The original contributions presented in the study are included in the article, further inquiries can be directed to the corresponding author.
